# Speckle-Tracking Echocardiography in Right Ventricular Function of Clinically Well Patients with Heart Transplantation

**DOI:** 10.3390/diagnostics14121305

**Published:** 2024-06-20

**Authors:** Xiang Ji, Junmin Zhang, Yuji Xie, Wenyuan Wang, Yiwei Zhang, Mingxing Xie, Li Zhang

**Affiliations:** 1 Department of Ultrasound Medicine, Union Hospital, Tongji Medical College, Huazhong University of Science and Technology, Wuhan 430022, China; jx1223908317@163.com (X.J.); m202376290@hust.edu.cn (J.Z.);; 2 Clinical Research Center for Medical Imaging in Hubei Province, Wuhan 430022, China; 3 Hubei Province Key Laboratory of Molecular Imaging, Wuhan 430022, China

**Keywords:** heart transplantation, echocardiography, speckle-tracking echocardiography, right ventricle, function

## Abstract

Heart transplantation (HT) is the mainstream therapy for end-stage heart disease. However, the cardiac graft function can be affected by several factors. It is important to monitor HT patients for signs of graft dysfunction. Transthoracic echocardiography is a simple, first-line, and non-invasive method for the assessment of cardiac function. The emerging speckle-tracking echocardiography (STE) could quickly and easily provide additive information over traditional echocardiography. STE longitudinal deformation parameters are markers of early impairment of ventricular function. Although once called the “forgotten ventricle”, right ventricular (RV) assessment has gained attention in recent years. This review highlights the potentially favorable role of STE in assessing RV systolic function in clinically well HT patients.

## 1. Introduction

Since its first use in 1967, heart transplantation (HT) has strikingly decreased the rates of mortality in patients with end-stage heart failure (HF) [1]. With a median survival rate of more than 12 years, HT is regarded as the definitive therapy for patients with end-stage HF [1,2,3]. Common postoperative complications of HT include acute rejection, infection, and graft heart failure [4,5,6], resulting in a poor prognosis. Among these, right ventricular (RV) failure is one of the most common postoperative complications [7,8]. It has been proposed that impaired RV function occurs after HT and is an independent predictor of adverse long-term outcomes [9]. A more marked decline in RV functional indices may transpire in the acute condition, as the proportional rise in RV afterload may be significantly more prominent in the pathological state than in the LV [10]. The efficacy of transplanted heart function may be affected by the complexity and diversity of donor–recipient relationships and surgical techniques. Several factors, including pericardiotomy surgery, injuries caused by ischemia–reperfusion, and progressive remodeling after HT, may lead to the difference in ventricular function between HT patients and health controls [11,12]. RV systolic function can be affected by pericardiotomy and transplanting, with longitudinal shortening being the most notable alteration [13]. The assessment of ventricular function is complicated, as transplanted ventricular performance may differ from that of healthy controls, even in clinically well-functioning HT patients [14,15]. HT causes imperceptible damage in the early postoperative stage, especially in the first stage after surgery, and significant changes in RV function may occur in patients with stable clinical symptoms. A recent study has also demonstrated that subclinical cardiac dysfunction may be an early indicator of cardiac injury rather than a component of the transplanted heart’s physiological adaption [16]. Therefore, an accurate characterization of RV function in follow-up studies of clinically well HT patients is essential for identifying changes in normal reference values and pathological values that require timely intervention, especially in patients with good clinical performance [17].

In recent years, there has been increasing interest in developing new diagnostic and prediction methods that could provide simple, accurate, fast, and inexpensive quantitative assessment of cardiac function to improve the ability to identify subclinical ventricular dysfunction in HT patients [18,19]. Because of its convenience and non-invasive nature, echocardiography is a particularly useful tool for assessing cardiac graft function [20,21,22,23,24]. However, considering the complexity of RV structure and contraction and the altered cardiac anatomy after HT, it is challenging to reveal subclinical cardiac impairment in HT patients by conventional echocardiography. Speckle-tracking echocardiography (STE) is a non-invasive, attractive, and angle-independent approach for measuring cardiac mechanical function by tracking the spatial motion of echo speckles within the myocardium in response to real-time motion and deformation of myocardial tissue [25,26,27,28,29,30,31]. STE evaluation of myocardium function was found to be a superior discriminator, which can differentiate the cardiac function between HT patients and normal individuals. Currently, two-dimensional (2D)—and three-dimensional (3D)—STE has been widely used to assess RV deformation, which could offer the possibility to better explore minor but significant changes in longitudinal myocardial function [15,32,33,34,35,36].

Unlike LV, RV free wall mainly consists of two layers of the myocardium: the superficial myocardium arranged in a circular pattern parallel to the atrioventricular groove, and the deep myocardium arranged in a neat longitudinal pattern from the atrioventricular groove toward the apex. The structure determines the characteristics of RV myocardial contraction, with the main contractile force coming from the longitudinal movement of the myofibers, which shortens the long axis of the RV by pulling the tricuspid annulus toward the apex [37]. Myocardial longitudinal strain (LS) is the magnitude of deformation in the long-axis direction of myocardial systolic and diastolic activity. RV longitudinal deformation has proved to be sensitive and robust for quantifying RV function [38]. Myocardial strain derived from STE could allow sensitive and accurate detection of early changes in RV function [14]. By comparing the changes occurring during serial evaluations, STE-derived RVFWLS and RVGLS are for diagnosing subclinical allograft dysfunction [39].

This paper reviews the efficacy of STE for assessing graft RV function in clinically well HT recipients, with a particular focus on the utility of RV longitudinal strain.

## 2. Comprehensive Evaluation of the Right Ventricle in Clinical Practice

Although cardiovascular magnetic resonance imaging (CMR) is considered the gold standard for evaluating RV function [40], the high cost and time-consuming nature of the procedure are barriers to its widespread use in clinical practice. By providing accurate information about cardiac geometry and ventricular contractility, echocardiography has emerged as the first-line imaging modality for assessing HT patients [41]. In accordance with the 2015 American Society of Echocardiography recommendations for cardiac chamber quantification, the following parameters were recommended for comprehensive evaluation of the RV systolic function in clinical practice: fractional area change (FAC), tricuspid annular plane systolic excursion (TAPSE), peak systolic velocity of tricuspid annulus, right-sided index of myocardial performance (RIMP), and 2D STE-derived RV strain [42].

## 3. STE

Accurate assessment of RV function by conventional echocardiographic methods continues to be challenging, given the complexity of RV structure and contraction. Advances in cardiac imaging and the development of new devices have led to increased availability of STE. The non-Doppler-based method of STE is a milestone that allows us to objectively quantify the myocardial strain in standard 2D and 3D images, with high accuracy, sensitivity, feasibility, and reproducibility [35,43,44]. This robust technique provides frame-by-frame tracking of natural acoustic markers, has less angle dependence than tissue Doppler imaging (TDI), and enables assessment of myocardial deformations in a multidirectional manner [45]. The application of STE in clinical settings has gradually increased, including ischemic heart disease, valvular heart disease, cardiomyopathy, etc. [46,47,48,49,50]. The European Association of Cardiovascular Imaging/Cardiovascular Imaging Department of the Brazilian Society of Cardiology suggested the use of STE in monitoring HT patients with clinical suspicion of function abnormalities [39]. Notably, the longitudinal strain has already proved to be a more robust, feasible, reproducible, and sensitive parameter for clinical dysfunction detection. STE-derived RV longitudinal strain measurement is known to be a more sensitive tool to unmask early and small changes in myocardial function, which is considered superior to conventional echocardiography [51], facilitating its clinical application in conditions analyzed in the following paragraphs. Additionally, studies showed that STE measurement of RV strain exhibited high consistency with that obtained by CMR in various cardiovascular diseases, and was more sensitive than RVEF measured on dynamic computed tomography (CT) in detecting RV systolic dysfunction in patients with significant regurgitation [52,53,54,55,56].

**Figure 1:**
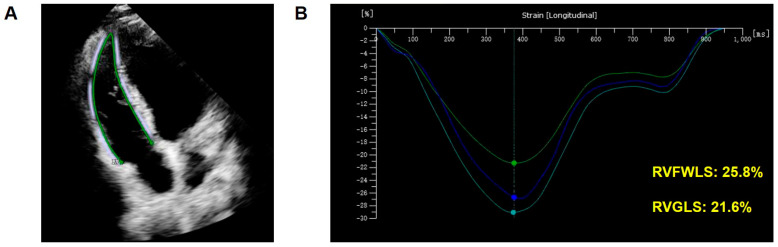
Example of assessment of RV systolic function by 2D-STE in a normal healthy person. (**A**) Tracing of 2D-RV longitudinal strain. (**B**) Processing of 2D-RV longitudinal strain. The green line represents the longitudinal strain of apical-free wall. The dark blue line represents the longitudinal strain of basal-free wall. The light blue line represents the longitudinal strain of mid-free wall. RV: right ventricular; 2D: two-dimensional; STE: speckle-tracking echocardiography.

**Figure 2:**
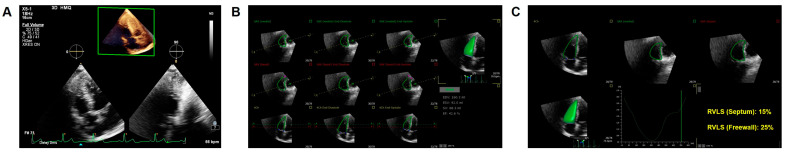
Example of assessment of RV systolic function by 3D-STE in a normal healthy person. (**A**) 3D image of RV-focused apical 4-chamber view. (**B**) RV endocardial border identification and tracking. (**C**) RV longitudinal strain. RV: right ventricular; 3D: three-dimensional; STE: speckle-tracking echocardiography.

STE longitudinal strain values, including RV global longitudinal strain (RVGLS) and RV free wall longitudinal strain (RVFWLS), were obtained from the analysis of three consecutive beats from the RV-focused apical four-chamber window [45] (Figure 1 and Figure 2).

**Figure 3:**
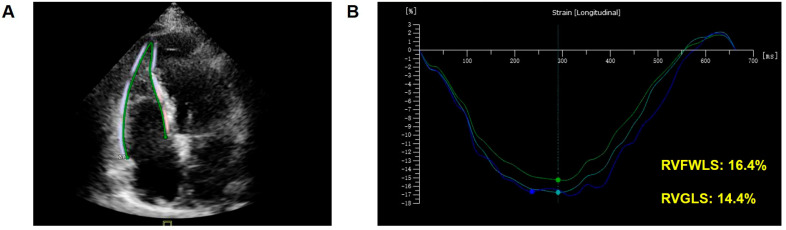
Example of assessment of RV systolic function by 2D-STE in a HT patient. (**A**) Tracing of 2D-RV longitudinal strain. (**B**) Processing of 2D-RV longitudinal strain. The green line represents the longitudinal strain of apical-free wall. The dark blue line represents the longitudinal strain of basal-free wall. The light blue line represents the longitudinal strain of mid-free wall. RV: right ventricular; 2D: two-dimensional; STE: speckle-tracking echocardiography; HT: heart transplantation.

**Figure 4:**
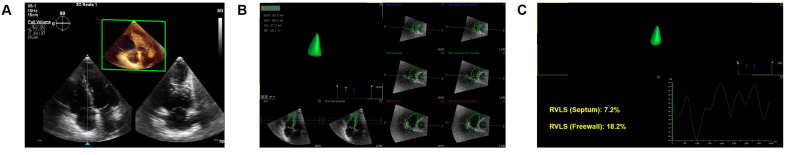
Example of assessment of RV systolic function by 3D-STE in a HT patient. (**A**) 3D image of RV-focused apical 4-chamber view. (**B**) RV endocardial border identification and tracking. (**C**) RV longitudinal strain. RV: right ventricular; 3D: three-dimensional; STE: speckle-tracking echocardiography; HT: heart transplantation.

Figure 3 and Figure 4 show STE measuring strain for detecting RV myocardial deformation in HT patients.

## 4. STE-Derived RV Strain in HT Patients

### 4.1. Associations between 2D-STE RV Strain and Other Hemodynamic Indices of the RV

In clinical practice, 2D-STE is currently the accepted approach for evaluating RV longitudinal strain (RVLS) because it has better consistency, less angular dependence, and higher feasibility than tissue Doppler strain and traditional echocardiography [10]. RV myocardial deformation decreased early after HT and improved progressively (without significant evidence) during long-term follow-up because of stable hemodynamics [57]. In patients undergoing HT, it is possible that the donor heart will recover from the effects of the peri hypertensive period as postoperative pulmonary artery pressures decrease, resulting in an improvement in overall RV function. In addition, patients at low risk for rejection are typically tapered to a low dose or discontinued from steroids entirely by 12 months after HT, which may also lead to a gradual improvement of RV longitudinal systolic function in non-rejection adult HT patients [58]. However, geometric changes in RV contractility may remain in HT patients, leading to persistent abnormalities in longitudinal contractility [59]. The use of immunosuppressive drugs has made diabetes, hypertension, and renal dysfunction frequent comorbidities following transplantation. The drugs adopted for long-term management of cardiac transplantation may present an important factor affecting long-term RV myocardial function post-HT [58]. Therefore, assessment of RV function is critical.

### 4.2. Current Findings Related to 2D- and 3D-STE RV Strain in HT Patients

Since STE’s introduction of strain imaging, which allows the detection of subtle changes in myocardial contractility with high reproducibility, echocardiographic imaging for serial surveillance of ventricular function in HT patients has become increasingly necessary. Studies have conducted in-depth longitudinal investigations of the progression of RV function utilizing 2D-STE in clinically well HT patients. And there has been a thorough description of how RV function changed following HT. Ingvarsson, A. et al. prospectively examined fifty clinically stable HT patients with transthoracic echocardiography and right heart catheterization and showed that the RV function parameters, including RVGLS and RVFWLS, were initially reduced and gradually significantly improved, at least during the first year following HT. The study confirmed that 2D-STE RV strain analysis seemed sensitive to graft surveillance, and the measurements of RVGLS and RVFWLS by 2D-STE demonstrated excellent reproducibility [17]. A study followed 31 clinically well HT patients and 25 healthy volunteers to evaluate the evolution of 2D-ventricular echocardiographic parameters in the first two years post-HT. The authors found the impairment of RVGLS and RVFWLS during early post-HT and detected the progressive improvement of the parameters until its complete normalization one year after HT. The work emphasized that it is helpful to monitor the evolution of RV function in HT patients using 2D-echocardiographic strain imaging, the excellent reproducibility of RVGLS measurement was also revealed by this study [25]. The prognostic value related to RV ventricular function changeability has been demonstrated in several studies [60,61,62,63,64,65]. The 2D-STE-obtained RV strain assessment has proved effective in offering prognostic information on HT patients. To evaluate the predictive value of RVFWLS measurement in low-risk patients at one year after HT, Barakat et al. retrospectively performed 2D-STE on 96 HT recipients free of antibody-mediated rejection or moderate to severe coronary allograft vasculopathy (CAV) (CAV, grade 2 to 3) at one year after transplantation. The study found that RV function parameters, such as fractional area change (FAC) and RVFWLS, were independently linked to unfavorable outcomes. It also showed that RVFWLS might be used in risk scores and future prospective studies for patient risk stratification following HT [66]. Due to the low volume of pediatric cardiac, research on pediatric HT patients is challenging. RV myocardial deformation by 2D-STE offered a new method for assessing graft RV systolic function in pediatric patients. To investigate the serial changes in RV systolic function in rejection-free children and young adults after HT, a study was performed using traditional and 2D-STE in ninety-six rejection-free HT patients. The work showed that all the RV function parameters were immediately impaired after HT and improved significantly over the first year. Moreover, except for FAC and myocardial performance index (MPI), tricuspid annular plane systolic excursion (TAPSE), peak systolic velocity of tricuspid annulus (S’), RVGLS, and RVFWLS remained abnormal by 1-year post-HT. The authors considered that the measurements of RVGLS and RVFWLS may improve the ability to accurately evaluate RV function in this special population [60]. Current findings regarding 2D-STE-derived RV strain assessment in clinically well HT patients are shown in Table 1.

The 2D-STE-derived myocardial strain can sensitively detect early changes in ventricular function and accurately quantify myocardial function [14]. However, the 2D plane of interest is not always visible during the cardiac cycle due to reduced field of view, geometric modeling, and speckle loss due to out-of-plane motion. Recently, the newly developed 3D-STE has been used to follow the myocardial strain of the RV. This technique, based on full-volume 3D data sets, has become a more physiologically sound tool for analyzing the complexity of RV mechanics. LV et al. validated the accuracy of 3D-STE in evaluating biventricular functions in a cohort of 35 HT patients who underwent both 3D echocardiography and CMR examination, and they compared 3D-STE-derived biventricular functions between 46 HT patients and 46 non-HT controls. The study demonstrated that the measurement of RVFWLS using 3D STE exhibited excellent reproducibility. The authors also found that 3D-STE showed excellent accuracy in assessing biventricular functions in HT patients against CMR, and RVFWLS measured by 3D-STE correlated well with CMR right ventricular ejection fraction (RVEF) (r = −0.83, *p* < 0.001). They indicated that even in clinically well HT patients, 3D biventricular mechanical functions were reduced. The study further provided normal 3D strain values of biventricular mechanical functions in HT patients and believed that the provided characteristics and values could be the basis for accurate assessment of ventricular dysfunction in follow-up examinations [14]. 3D-STE RV strain assessment could also provide a new and accurate approach for the serial assessment of HT patients. Ran, H. et al. performed 3D-STE at 1-, 5-, and 10-year follow-ups in 62 stable HT patients, together with routine echocardiographic evaluation in 32 control subjects, and described the reduced RV deformation. Moreover, they also found that RV deformation decreased more than that of the LV. The study showed that strain values could provide more sensitive detection of ventricular dysfunction than traditional measurements in HT patients [67]. In a study that included 58 clinically well adult HT patients and 58 healthy controls. The authors performed conventional and 3D-STE in all HT patients at 1, 3, 6, 9, and 12 months post-HT. By using 3D-RVFWLS, the significant improvement of RV systolic function over time in clinically well adult HT patients had been detected. They further demonstrated that RVFWLS of HT patients remained lower than the control, even by 12 months post-HT. The work indicated that 3D-STE may provide a suitable method for the follow-up of RV systolic function in HT patients, and demonstrated that the aggressive treatment may play an important role in affecting the performance of the right ventricle in HT patients [58]. Like 2D-STE, 3D-STE has also been applied to reveal subclinical impaired RV function in children and young adults without acute rejection. Except for the adult patients, LV et al. enrolled 30 clinically well pediatric HT patients and 30 healthy controls to evaluate the biventricular function of pediatric HT patients by 3D-STE. They showed that compared with the control group, RVFWLS was significantly lower in the HT group. Moreover, RVFWLS weakly correlated with postoperative pulmonary artery systolic pressure and preoperative mean pulmonary artery pressure. The authors thought that strain values of 3D-STE could help subsequent studies in pediatric HT patients [68]. Similarly, Chinali et al. evaluated the RVGLS in 60 patients with HT performed at pediatric age with apparently normal cardiac function. A significant net reduction in RVGLS was demonstrated in this study, and the authors confirmed that even in the absence of acute rejection and in the presence of a normal ejection fraction, HT patients have RV systolic dysfunction by detection of 3D-STE [69].

**Table 1:** Current findings regarding 2D-STE RV strain assessment in clinically well patients with heart transplantation.

References	Sample Size	RV Strain Parameters	Software	Main Findings
Moñivas et al. 2016 [25]	31	RVGLS	X-Celera version 7.0, Philips	RVGLS significantly reduced early after HT and improved progressively until its complete normalization 1-year post-HT. The reproducibility of RVGLS measurement was excellent.
Chinali et al. 2017 [69]	60	RVGLS	Q-LAB 10 version, Philips Medical System, Andover, MA, USA	A significant reduction in RVGLS existed in transplanted hearts even in the absence of signs of graft failure and in the presence of a normal ejection fraction.
Barakat et al. 2017 [66]	96	RVFWLS	EchoPAC Version 113, General Electric Medical Systems	RVFWLS was independently associated with incident rejection, CAV, and death in low-risk patients 1 year after HT. The measurement of RVFWLS by 2D-STE showed good to excellent reproducibility.
Harrington et al. 2019 [59]	96	RVGLS RVFWLS	Tomtec, Chicago, IL, USA	RVGLS and RVFWLS immediately reduced after HT and improved significantly over the first year. The RV deformation abnormalities persisted at 1 year post-HT. The measurements of RVGLS and RVFWLS by 2D-STE showed good to excellent reproducibility.
Ingvarsson et al. 2021 [17]	50	RVGLS RVFWLS	CMQ, Q-lab 10.3, Philips iE33, Philips Healthcare, Eindhoven, NL, USA	The RV strain parameters, especially the RVFWLS, gradually improved during the first year following HT. The measurements of RVGLS and RVFWLS by 2D-STE had excellent reproducibility.
Li et al. 2023 [58]	58	RVFWLS	Tomtec 2D strain software, 2D Cardiac Performance Analysis, Germany	In clinically well adult HT patients, 2D-RVFWLS increased with significance from 1 to 6 months post-HT. 2D-RVFWLS was significantly lower in HT patients by 12 months post-HT than in healthy controls. The intraobserver and interobserver ICCs of 2D-RVFWLS were high.
Li et al. 2023 [70]	155	RVFWLS	Tomtec 2D strain software, 2D Cardiac Performance Analysis, Germany	2D-STE-derived RVFWLS was an independent predictor of adverse outcomes for HT patients. The intraobserver and interobserver ICCs of 2D-RVFWLS were high.

RV, right ventricular; RVFWLS, right ventricular free wall longitudinal strain; RVGLS, right ventricular global longitudinal strain; 2D, two-dimensional; STE, speckle-tracking echocardiography; CAV, coronary allograft vasculopathy; HT, heart transplantation; ICCs, intraclass correlation coefficients.

### 4.3. Follow-Up of 3D-STE RV in Risk Stratification of Patients after HT

The application of 3D-STE for further risk stratification in HT patients has been explored and validated. The evaluation of RV function using this technique has been shown to improve the prognostic assessment of these patients. Based on the previous study, Li et al. further investigated the prognostic value of 3D-RVFWLS in adult HT patients. They confirmed that HT patients with adverse events had lower RVFWLS than those without adverse events and indicated that 3D-RVFWLS was the strongest independent predictor of adverse outcomes in adult HT patients. The authors considered that 3D-RVFWLS may be recommended for risk stratification in HT patients [70]. Current findings regarding 3D-STE-derived RV strain assessment in clinically well HT patients are shown in Table 2.

In summary, STE longitudinal strain analysis is more sensitive than conventional echocardiography for detecting abnormalities of RV systolic function. It could reflect subtle changes in RV function and improve understanding of cardiac mechanics in such a population. The technique could also enhance the strain values to sensitively monitor the evolution of RV systolic function with high reproducibility during the follow-up of HT patients. Moreover, 3D-STE strain measurement has been demonstrated to provide an accurate evaluation of RV function when compared with the referenced standard CMR in these patients. What is more, the impaired STE RV longitudinal deformation is associated with poor prognosis, assessment of STE RV strain is a suitable method for projecting the prognosis of this unique population.

## 5. Current Limitations and Future Prospects

First, although some studies considered transplanted patients, the small sample size of the population might reduce the impact of the research and the overall conclusions of the authors, future more extensive multi-center and large-sample studies are warranted to verify the findings. Second, further research is also needed to confirm the prognostic value of STE strain parameters in such a population, as the number of studies focusing on this was relatively small. Third, the primary limitation of STE strain analysis is its intrinsic requirement for good picture quality, which has not been adequately addressed thus far [71,72,73,74,75]. Additional research is necessary to maximize its clinical application.

Although STE RV strain assessment of HT patients is still limited by some issues, it could become a valid method for these patients in the near future. For a definitive standardization of the use, future experts’ consensus to identify reference values of RV strain parameters in HT patients is highly expected. Future research should also consider combining STE with multimodal evaluation and artificial intelligence (AI). Establishing a multimodal evaluation and predictive model for HT patients through a multi-center study, as well as integrating other imaging examinations and clinical data with large samples, is a direction worth exploring. With the continued development of AI technology, STE will play a more critical role in clinical applications for HT patients.

## 6. Conclusions

Prior to the development of echocardiography, it was challenging to diagnose subclinical systolic dysfunction in the RV. However, with the introduction of STE, this problem has been successfully solved. STE has proven helpful in detecting and evaluating minor abnormalities in RV systolic function that conventional techniques may not easily detect. In addition, STE monitors longitudinal changes in the RV, allowing clinicians to assess the progression or regression of dysfunction continuously. Harnessing the power of STE helps physicians identify subclinical systolic dysfunction in the RV and provides essential information for prognostic evaluation in clinically well patients with HT.

## Figures and Tables

**Table 2:** Current findings regarding 3D-STE RV strain assessment in clinically well patients with heart transplantation.

References	Sample Size	RV Strain Parameters	Software	Main Findings
Lv et al. 2020 [14]	81	RVFWLS	TomTec Imaging Systems, Unterschleissheim, Germany	3D-STE can accurately assess RV function of HT patients. RVFWLS was reduced in clinically well HT patients. The measurement of RVFWLS by 3D-STE had excellent reproducibility. 3D-STE RVFWLS correlated well with CMR-RVEF (r = −0.83, *p* < 0.001).
Ran et al. 2020 [67]	62	RVGLS Strain RV basal average Strain RV mid average Strain RV apical average	Toshiba Medical Systems Corp	RVGLS reduced after HT. HT patients had stable but lower RVGLS with long-time follow-ups. RVGLS obtained by 3D-STE could more sensitively reveal dysfunction than conventional echocardiographic measurements in HT patients.
Lv et al. 2020 [68]	30	RVFWLS	4D RV-Analysis 2.0, TomTec Imaging Systems	The RVFWLS was reduced in clinically well pediatric HT patients. The measurement of RVFWLS by 3D-STE showed good to excellent reproducibility.
Li et al. 2023 [58]	58	RVFWLS	Tomtec 4D Cardio-View software, 4D RV-Analysis 2.0, Germany	In clinically well adult HT patients, 3D-RVFWLS improved significantly over time, even up to 12 months post-HT. 3D-STE-derived RVFWLS is the most suitable method for the evaluation of RV systolic function. The intraobserver and interobserver ICCs of 3D-RVFWLS were high.
Li et al. 2023 [70]	155	RVFWLS	Tomtec 4D Cardio-View software, 4D RV-Analysis 2.0, Germany	3D-STE-derived RVFWLS was the strongest independent predictor of adverse outcomes for HT patients. The intraobserver and interobserver ICCs of 3D-RVFWLS were high.

RV, right ventricular; RVFWLS, right ventricular free wall longitudinal strain; RVEF, right ventricular ejection fraction; RVGLS, right ventricular global longitudinal strain; 3D, three-dimensional; STE, speckle-tracking echocardiography; HT, heart transplantation; CMR, cardiac magnetic resonance; ICCs, intraclass correlation coefficients.
